# ComplexViewer: visualization of curated macromolecular complexes

**DOI:** 10.1093/bioinformatics/btx497

**Published:** 2017-08-03

**Authors:** Colin W Combe, Marine (Dumousseau) Sivade, Henning Hermjakob, Joshua Heimbach, Birgit H M Meldal, Gos Micklem, Sandra Orchard, Juri Rappsilber

**Affiliations:** 1 Wellcome Trust Centre for Cell Biology, School of Biological Sciences, University of Edinburgh, Edinburgh, UK; 2 European Bioinformatics Institute (EMBL-EBI), European Molecular Biology Laboratory, Wellcome Genome Campus, Cambridge, UK; 3 Cambridge Systems Biology Centre, University of Cambridge, Cambridge, UK; 4 Department of Genetics, University of Cambridge, Cambridge, UK; 5 Chair of Bioanalytics, Department of Bioanalytics, Institute of Biotechnology, Technische Universität Berlin, Berlin, Germany

## Abstract

**Summary:**

Proteins frequently function as parts of complexes, assemblages of multiple proteins and other biomolecules, yet network visualizations usually only show proteins as parts of binary interactions. ComplexViewer visualizes interactions with more than two participants and thereby avoids the need to first expand these into multiple binary interactions. Furthermore, if binding regions between molecules are known then these can be displayed in the context of the larger complex.

**Availability and implementation:**

freely available under Apache version 2 license; EMBL-EBI Complex Portal: http://www.ebi.ac.uk/complexportal; Source code: https://github.com/MICommunity/ComplexViewer; Package: https://www.npmjs.com/package/complexviewer; http://biojs.io/d/complexviewer. Language: JavaScript; Web technology: Scalable Vector Graphics; Libraries: D3.js.

## 1 Introduction

A wide variety of experimental techniques are used to investigate the molecular interactions that drive and regulate cellular processes. For example, a gel filtration experiment identifies groups of associated proteins—these are not necessarily binary interactions, and to expand them into multiple binary interactions for the purpose of network visualization introduces false positives. ComplexViewer avoids the need for this expansion and provides a more biologically realistic representation of the interaction.

The IMEx Consortium (Orchard *et al.*, 2012) is an international collaboration designed to systematically capture published interaction data in publicly available databases. The IMEx Consortium uses the Proteomics Standards Initiative-Molecular Interaction (PSI-MI) data standards to enable the reuse and exchange of data (Hermjakob *et al.*, 2004; Kerrien *et al.*, 2007). These standards capture a high level of detail to allow a dynamic view of the cellular environment, for example, recording n-ary not just binary interactions. However, no viewer exists which can support all the information curated into an IMEx record.

The European Bioinformatics Institute (EMBL-EBI) has developed the Complex Portal (http://www.ebi.ac.uk/complexportal), an encyclopaedia of manually curated macromolecular complexes that captures the biologically functional units of proteins and other participating molecules by amalgamating experimental information and assorted background information (Meldal *et al.*, 2015). The Complex Portal data are stored in the IntAct database (Kerrien *et al.*, 2012; http://www.ebi.ac.uk/intact) and conform to PSI-MI standards.

The Complex Portal website requires a visualization that can accurately represent the following aspects of PSI-MI data: interactions with more than two participants (n-ary interactions); sequence features relevant to the interaction, such as binding domains; a range of biomolecules as interactor types (proteins, small molecules, nucleic acids); discontinuous sequence features; and, stoichiometry information. ComplexViewer meets these needs and provides 2D diagrams of the topology of the rapidly increasing number of complexes described by this resource.

## 2 Implementation

PSI-MI data standards come in two distinct formats—XML based and tab-delimited. The tab-delimited format is not a suitable basis for the ComplexViewer because (i) it removes the information on how specific binding regions interact, and (ii) cannot record interactions with more than two participants. The XML format fully captures the detail of n-ary interactions but is not designed for parsing within browsers. To provide this detail in a concise, easy-to-parse format we designed the JavaScript Object Notation format MI-JSON. At the 2017 HUPO PSI, the Molecular Interaction workgroup decided MI-JSON will be its recommended protocol for serving interaction data to web pages and visualization tools. MI-JSON can be generated from any PSI-MI compliant data source using the Java Molecular Interactions library (JAMI, https://github.com/MICommunity/psi-jami, manuscript in preparation).

ComplexViewer is derived from xiNET (Combe et al., 2015), which already contained functionality for displaying pairs of linked residues but could not display binding regions nor support n-ary interactions.

## 3 Results and discussion

**Fig. 1:**
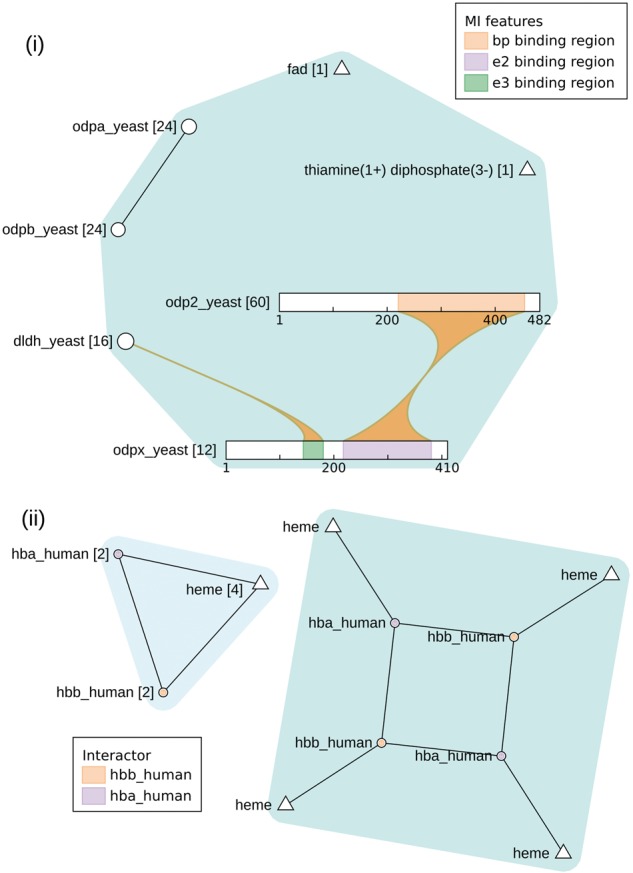
(**i**) EBI-9691559: Mitochondrial pyruvate dehydrogenase complex. The complex (in blue) is a single n-ary interaction in which interactions between certain participants are known and some binding sites are known. Stoichiometry information is given in square brackets. (**ii**) EBI-9008420: Haemoglobin HbA complex. This complex is shown both with the stoichiometry information collapsed (left) and expanded (right)

ComplexViewer can display macromolecular complexes of varying size while preserving their internal topology, binding features and stoichiometry. Figure 1 shows two examples from the EMBL-EBI Complex Portal.

The Mitochondrial pyruvate dehydrogenase complex is one of the largest structures in the Complex Portal. Displaying its 136 individual protein molecules would overwhelm the view and therefore for complexes in which any participant has a stoichiometry of more than 30, the stoichiometry is collapsed and displayed in square brackets. This example also highlights the varying levels of information available for the internal topology, with detailed binding features available for LAT1 (odp2) and PDX1 (odpx). The Haemoglobin HbA complex is much smaller which allows ComplexViewer to display it with its stoichiometry expanded and a clearly defined internal topology.

In addition to the Complex Portal, ComplexViewer has been incorporated into HumanMine (Smith *et al.*, 2012; http://www.humanmine.org) and YeastMine (Balakrishnan *et al.*, 2012; http://yeastmine.yeastgenome.org), which are data warehouses of model organism information, and also into the IntAct Editor (Orchard *et al.*, 2014; https://github.com/EBI-Intact/intact-editor). The IntAct Editor is a tool designed to assist with the curation process; providing a more detailed visualization has enabled more accurate and complete curation.

## 4 Conclusion

ComplexViewer is proving a useful tool for visualizing molecular interaction data. We intend to extend its functionality to show the hierarchical nesting of complexes and the full range of PSI-MI data for curated experimental evidence, such as is stored in IntAct (Kerrien *et al.*, 2012). The main challenge in extending the applicability of ComplexViewer will be allowing the navigation of larger networks, comprised of many such n-ary interactions. We think achieving this goal would be a beneficial step away from the n-ary to binary expansion procedures which, in essence, add false data prior to visualization.

## Funding

This work was supported by the Wellcome Trust [103139 to J.R., 063412 to H.H., J.R.]; and the BBSRC [BB/L024179/1 to S.O., G.M. and H.H.]. Wellcome Trust Centre for Cell Biology is supported by core funding from the Wellcome Trust [203149].

*Conflict of Interest*: none declared.
